# Predictors of postpartum modern contraceptive use intentions among pregnant women in urban Ghana: a cross-sectional study

**DOI:** 10.11604/pamj.2026.53.13.42783

**Published:** 2026-01-12

**Authors:** Joseph Lasong, Yula Salifu, Ahmad Sukerazu Alhassan

**Affiliations:** 1 Department of Population and Reproductive Health, School of Public Health, University for Development Studies, Tamale, Ghana

**Keywords:** Postpartum, urban, Ghana, family planning, contraceptive use

## Abstract

**Introduction:**

the provision of modern family planning services is crucial for postpartum women. In some cases, women have become pregnant before having their first menstruation following a delivery. Often, such pregnancy is deemed unplanned and unwanted and may be aborted. Therefore, identifying the predictors of extended postpartum contraceptive use among women is essential for efficient postpartum contraceptive use.

**Methods:**

a health facility-based, cross-sectional study was conducted at the Ashanti Regional Hospital, Kumasi, Ghana in 2018. A sample size of 298 pregnant women attending the antenatal clinics was used for the study. A 55-item questionnaire was used to collect data via a simple random sampling technique and multiple logistic regression, Pearson´s Chi-square, and descriptive statistics were performed using IBM-SPSS (v22.0).

**Results:**

the magnitude of postpartum modern contraceptive use intention was 30.20% among pregnant women. The most frequent method of intended use was oral contraceptives (18.8%). In the multivariate logistic regression model, we found that urban residence [AOR = 6.40, 95% CI: (1.22-24.66)], history of unintended pregnancy [AOR = 3.16, 95% CI: (1.02-9.80)] and frequency of sexual activity-once a month [AOR = 3.59, 95% CI: (1.23-10.53)], were factors positively associated with post-partum contraceptive use intentions among pregnant women.

**Conclusion:**

the use of extended postpartum contraceptive services was not significant among the studied respondents. Place of residence, mass media, history of unintended pregnancy, and frequency of sexual activity once a month were important predictors of intentions to use modern contraceptives among urban Ghanaian women.

## Introduction

Modern family planning methods save mothers' and children´s lives and promote health and well-being [1,2]. It saves lives by promoting birth spacing and limiting maternal age for childbearing [3,4]. If women waited 24 months or more after delivery to become pregnant again, the lives of 1.8 million children under age five could be saved annually [5]. Children born within less than two years of the spacing interval are two times more likely to die in the first year of life than those born after at least two years [4, 6-8]. Family planning use is also a basic fundamental fulfillment of women´s rights to choose when and how many children to have [9]. This helps to promote women's empowerment in a way that women can make independent decisions about their health and modern family planning use. Thus, this helps to achieve Sustainable Development Goal five (SDG-5), which aims at achieving gender equality and empowering all women and girls [1, 10-12]. The postpartum period begins immediately after the child is born. Six weeks after delivery, a woman´s physiology returns to normal, and she can resume an active sexual life [13,14]. This puts every mother, even those who are breastfeeding exclusively, at risk of another pregnancy. The majority of these women are unprepared to become pregnant again, yet most new mothers seldom use family planning to prevent such unwanted pregnancies. The majority of such women are from low or middle-income countries [12,15]. Family planning uptake during the postpartum period has the potential to significantly reduce 71% of unintended pregnancies (53 million unintended pregnancies), 22 million fewer unwanted births, 25 million induced abortions, and 7 million miscarriages [2,3]. Hence, every mother needs to initiate family planning use at six weeks postpartum. It is estimated that in 2012, there were more than 74 million unintended pregnancies in the developing countries, with 4.6 million reported in West Africa alone [1,16]. Ghana recorded one of the highest levels of unmet need for family planning (36 %) among married women in 2008 [17]. Yet modern contraceptive use was higher (17 %) than in 20 other African countries. It may be that high unmet need in Ghana is a partial reflection of women´s growing tendency to articulate a need for birth spacing or limiting birth intention [18-20]. This study aimed to assess factors of extended postpartum family planning use among women in urban Ghana. Addressing the specific factors associated with extended postpartum family planning use among women in urban Ghana will help in prioritizing interventions. This is of greater significance for the proper design, implementation, and evaluation of programs, for a more effective and efficient family planning towards achieving sustainable development goals by reducing maternal and child mortalities.

## Methods

**Study design:** a health facility-based, cross-sectional study was conducted at the Ashanti Regional Hospital in Kumasi, Ghana for 2 weeks in 2018.

**Study setting:** the study area Kumasi in the Ashanti Region, is a multicultural city with diverse clans or ethnic groups in Ghana and other nationals, which makes it suitable for policy involvement [21]. Kumasi is the second-largest city in Ghana and the administrative capital of the Ashanti Region. It has an estimated population of 1,581,141 with an annual growth rate of 3.4%. Males constitute 49% and females 51% [21]. Politically, the metropolis is divided into ten (10) sub-metros. The city is an active commercial, academic, and recreational epicenter with vibrant logging and lumber industries, general commerce, and an automobile repair industry. It also has major physiognomies of Ghana, and thus, the results from the study are extrapolated to characterize what pertains in most Ghanaian societies. The Ashanti Regional Hospital is situated in the Asokwa sub-metro in the Kumasi metropolis but extends its services beyond the sub-metro to the surrounding districts [21].

**Study participants:** study participants eligible for the study were pregnant women aged 15- 49 years living in Ashanti Region attending antenatal care at the Regional Hospital (Kumasi) in the Kumasi Metropolitan Assembly from August to September 2018 and not diagnosed with any cognitive impairment. All pregnant women aged 15-49 years who were living in the Ashanti Region and accessing health services at the Ashanti Regional Hospital at the time of the study were included in the study. Only those eligible, not diagnosed with cognitive impairment, and who were willing to participate in the study were selected. Women who fell within the category but were not willing to take part in the study were excluded. Furthermore, pregnant women who are not living in the Ashanti Region were excluded from the study.

**Study variables:** firstly, the definition of “intention to use a family planning method” was addressed and asked as “intending to use any modern contraceptive method in the immediate and extended postpartum period”. Subsequently, women were asked whether they had an intention to use a modern contraceptive method (yes or no). Modern contraceptive methods refer to safe, effective, and legal methods to prevent pregnancy, such as the pill, implants/Norplant, injectable, condoms, the intra-uterine device (IUD), and male sterilization. The independent variables were selected based on their plausibility in reference to previous studies. These include demographics such as age, marital status, residence, history of abortion, frequency of contraceptive use, attitude towards contraceptive use, history of unintended pregnancy, frequency of sexual activity, contraceptive use at first sexual intercourse, and source of contraceptive knowledge.

**Data sources and measurements:** the questionnaires were adopted and adapted from studies [2-4,17-20] and expert reviews. A simple random sampling method was used in selecting respondents to ensure that each respondent in the sampling frame had an equal chance of being included or excluded from the sample. Simple random sampling is a probability sampling procedure that ensures that every individual unit in the population under investigation has a chance of being selected into or excluded from the study sample. The ballot method, which is a method of simple random sampling, was employed. Potential study participants were briefed on the rationale of the study and were asked to sign a consent form when they agreed to take part. They were selected at random by picking from a box containing pieces of paper with numbers. These pieces of paper were collected back from them, and their respective numbers were noted against their names. The papers were put into a container and thoroughly mixed, and then one by one at random until the 25 desired sample size was achieved for the particular day. Structured questionnaires for the study were administered to these selected respondents by trained assistants. Any respondent who refused to take part in the study was skipped, and a different number was picked from the box. This process was done on each day until the calculated sample size of 298 for the study was obtained after 12 working days. Research assistants were trained to ensure the effective collection of data. Field editing of questionnaires was conducted by the supervisor on a daily basis to check for correctness and completeness. Double data entry was adopted.

**Bias:** the chances of self-reporting bias cannot be ignored while interpreting the findings. Again, social desirability bias could occur due to the sensitive nature of sexual activity and contraception. The health facility-based population is limited and may not reflect the postpartum contraceptive use across the country.

**Quantitative variables:** to determine the magnitude of contraceptive use intentions postpartum (CUIP), the dependent variable was categorized as intentions (yes and no). Independent variables were categorized into groups, and predictors of CUIP were identified using odds ratios.

**Study size:** a sample size of 298 participants was estimated for the study using the Kish-Leslie formula, with a contraceptive prevalence rate of 23 % (Population Reference Bureau, 2013). A 55-item questionnaire was used to collect data via a simple random sampling technique.

$$N=\frac{Z^{2*}P\left(1-P\right)}{e^{2}}$$

Where N = sample size required; Z is the confidence level (95% level of confidence-1.96); P is the reported national prevalence of contraceptive use and e is the margin of error (5% =0.005).

**Statistical methods:** the IBM Statistical Package for Social Sciences (SPSS) version 22.0 was used for analyses after a double data entry and cleaning was done using Epi Info 3.5.1. The statistical analysis focused on 298 pregnant women of reproductive age 15-49 years. Frequency and percentages for descriptive statistics and Chi-square test for the association between the dependent and independent variables were performed. Additionally, crude and adjusted binary logistic regression was used to pinpoint the main correlates of postpartum modern contraceptive use. A test of statistical significance at p < 0.05 was employed for all analyses.

**Ethical approval and consent to participate:** ethical clearance was acquired from the Committee on Human Research Publication and Ethics of the Kwame Nkrumah University of Science and Technology (CHRPE/AP/546/18). Approval was also obtained from the Ashanti Regional Hospital and the various heads of departments before the study. The aim and process of the study were well explained to the participants to obtain their consent and the right to discontinue at any time. They were also guaranteed confidentiality and privacy.

## Results

**Participants and descriptive data:** a total of 298 participants were involved in the study. Table 1 shows the distribution of selected sociodemographic characteristics and contraceptive knowledge among women of childbearing age. The majority of respondents (68.50%) were adults with ages greater than 25 years. Most participants (62.10%) were married, 83.2% were from urban areas, 55.4% had a history of abortion, and 59.1% had a history of unintended pregnancy. About 45.30% of them rated the frequency of contraceptive use as ‘occasionally´. Approximately two-thirds of the respondents (64.3%) had an unfavorable attitude towards contraceptive usage, 65.8% had sexual activity once a week, and about three-fourths (72.8%) never use contraceptives at first sexual intercourse. Regarding sources of contraceptive knowledge, 37.6% of participants accessed advice from family and friends, 30.5% from health professionals, 25.8% from mass media, and only 6% from course education.

**Table 1:** demographic characteristics of pregnant women aged 15-49 years in Kumasi, Ghana, 2018

	Post-partum modern contraceptive use intentions				
**Variables**	**Total = 298**	**No = 208 (69.8 %)**	**Yes= 90 (30.2 %)**	**χ2**	**P-value**
	N (%) *	N (%) **	N (%) **		
**Age†**				2.321	0.128
Early adult (<25)	94 (31.50)	60 (63.8)	34 (36.2)		
Late adult (>25)	204 (68.50)	148 (72.5)	56 (27.5)		
**Marital status**				6.936	0.008
Married	185 (62.10)	119 (64.3)	66 (35.7)		
Never married	113 (37.90)	89 (78.8)	24 (21.2)		
**Types of residence**				4.243	0.039
Rural	50 (16.80)	41 (82.0)	9 (18.0)		
Urban	248 (83.20)	167 (67.3)	81 (32.7)		
**Religion†**				0.213	0.645
Christian	280 (94.3)	196 (70.0)	84 (30.0)		
Muslim	17 (0.3)	11 (64.7)	6 (35.3)		;
**Respondent's educational level**				3.260	0.071
Never attended	29 (9.7)	16 (55.2)	13 (44.8)		
Attended	269 (90.3)	192 (71.4)	77 (28.6)		
**Do you access contraceptives in your area**				4.678	0.031
No	270 (90.9)	194 (71.9)	76 (28.1)		
Yes	27 (9.1)	14 (51.9)	13 (48.1)		
**Ever had abortion**				18.986	<0.001
No	133 (44.60)	110 (82.7)	23 (17.3)		
Yes	165 (55.40)	98 (59.4)	67 (40.6)		
**Respondent’s employment status**				0.043	0.837
Unemployed	234 (78.5)	164 (70.1)	70 (29.9)		
Employed	64 (21.5)	44 (68.8)	20 (31.2)		
**Source of contraceptive knowledge †**				22.141	<0.001
Mass media	77 (25.80)	46 (59.7)	31 (40.3)		
Family and friends	112 (37.60)	68 (60.7)	44 (39.3)		
Health professionals	91 (30.50)	79 (86.8)	12 (13.2)		
Course education	18 (6.10)	15 (83.3)	3 (16.7)		
**Frequency of contraceptive use†**				189.058	<0.001
Never	108 (36.20)	23 (21.3)	85 (78.7)		
Occasionally	135 (45.30)	131 (97.0)	4 (3.0)		
Always	55 (18.50)				
**Attitude to contraceptive use**		54 (98.2)	1 (1.8)	44.575	<0.001
Negative/unfavorable	191 (64.30)	159 (83.2)	32 (16.8)		
Positive/favorable	106 (35.70)				
**Had unintended pregnancy before**		49 (46.2)	57 (53.8)	19.276	<0.001
No Yes	121 (40.90)	102 (84.3)	19 (15.7)		
Yes	175 (59.10)				
**Frequency of sexual activity**		106 (60.6)	69 (39.4)	5.978	0.014
Once a week	196 (65.80)	146 (74.5)	50 (25.5)	;	
Once a month	102 (34.20)				
**Contraceptive use at first sexual intercourse**		62 (60.8)	40 (39.2)	44.276	<0.001
No	217 (72.80)	128 (59.0)	89 (41.0)		
Yes	81 (27.20)	80 (98.8)	1 (1.2)		

† : Re-categorised *: column percentage, **: row percentage. p ≤ 0.05

### Outcome data

**Figure 1:**
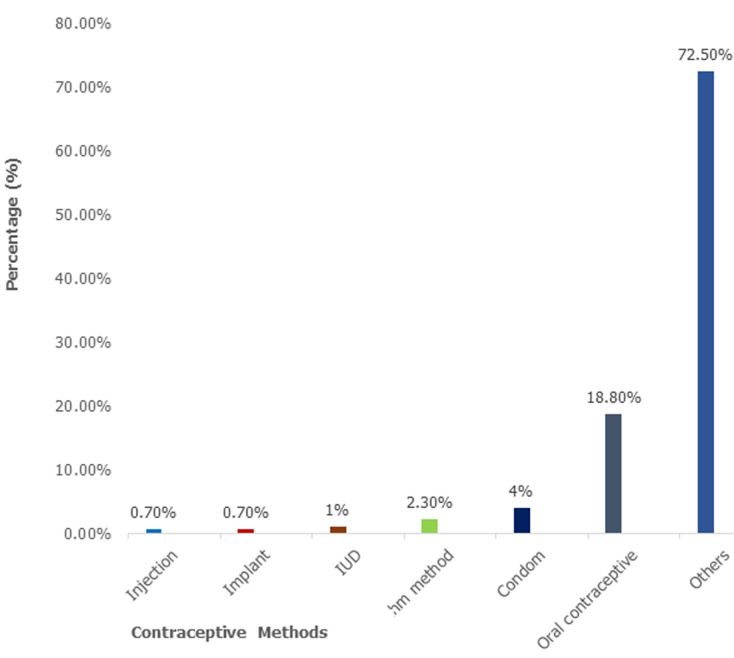
future contraceptive methods usage among pregnant women in Kumasi metropolis, 2018

**Intended contraceptive usage after delivery:** the magnitude of postpartum modern contraceptive use intention was 30.20% among pregnant women. Frequency distribution of the future contraceptive usage by type indicates that the majority (72.5%) of the women respondents may not use any contraceptive method in the future (Figure 1). Almost one-fifth (18.8%) of the women chose to use oral contraceptives, while condoms (4.0%), rhythm method (2.3%), IUD (1.0 %), implant (0.7%), and injectables (0.7%) were selected for future use by respondents, respectively.

### Main results

**Predictors of postpartum contraceptive use intentions:** to assess the magnitude and direction of the relationship between the intention to use contraceptives, sexual and reproductive demographic characteristics, bivariate logistic regression was used. At the bivariate level, respondents who never-married women were 51% less likely to have the intention to use contraceptives than married women (COR = 0.49, 95% CI: 0.28, 0.84; Table 2). Women residing in urban areas had 2.21 times more intention to use contraceptives than rural residents (COR = 2.21, 95% CI: 1.02, 4.77). Women who had a history of abortion (COR = 3.27, 95% CI: 1.89, 5.65), and unintended pregnancy (COR= 3.49, 95% CI: 1.96, 6.22) were found to be more likely to use contraceptives than their counterparts. Being an occasional (COR = 0.01, 95% CI: 0.00, 0.04) or regular (COR = 0.01, 95 % CI: 0.00, 0.03) user of contraceptives had a protective effect towards intention to use contraceptives. Women who used contraceptives at their first sexual intercourse were found to have 98% less (COR = 0.02, 95% CI: 0.00, 0.13) intention to use contraceptives than those who never used contraceptives. Women with a positive attitude in contraceptive usage had 5.78 times more intention to use contraceptives than women with a negative attitude towards contraceptive usage (COR = 5.78: 95% CI: 3.37, 9.90). Women who had a sexual activity once a month (COR = 1.88, 95% CI: 1.13, 3.14) were more likely to use contraceptives than women who had sexual activity once a week. Those who gained contraceptive knowledge from mass media were also more likely to have intentions to use contraceptives as compared to those who gained their knowledge from health professionals (COR = 0.23, 95 % CI: 0.11, 0.48) and course education (COR 0.30, 95% CI: 0.08, 1.11). However, women who use mass media as a source of contraceptive knowledge recorded no significant difference in their intention to use contraceptives as compared to women who had family and friends as sources of contraceptive information (COR = 0.96, 95% CI: 0.53, 1.74; Table 2).

**Table 2:** bivariate and multivariate analysis of the predictors of modern contraceptive use intention in Ghana, 2018

	Post-partum modern contraceptive use intentions						
Variables	Crude			Adjusted			
				AOR			
Marital status	OR	95 % CI	P-value		95 % CI	P-value	
Married	1	1		1	1		
Never married							
**Types of residence**	0.49	0.28-.0.84	<0.001	0.31	0.11-0.87	0.027	
Rural	1	1		1	1		
Urban							
**Ever had abortion**	2.21	1.02-4.77	0.040	5.71	1.52-21.42	0.010	
No	1	1		1	1		
Yes	3.27						
**Frequency of contraceptive use**		1.89-5.65	<0.001	0.22	0.05-0.85	0.029	
Never	1	1		1	1		
Occasionally	0.01	0.00-0.04	<0.001	1.53	0.13-0.08	17.47	
Always	0.01						
**Attitude to contraceptive use**		0.00-0.03	<0.001	176.05	15.16-2044.39	<0.001	
Negative	1	1		1	1		
Positive	5.78						
**Had unintended pregnancy before**		3.37-9.90	<0.001	1.33	0.49-3.57	0.575	
No Yes	1	1		1	1		
	3.49						
**Frequency of sexual activity**		1.96-6.22	<0.001	2.85	0.94-8.68	0.065	
Once a week	1	1		1	1		
Once a month	1.88	1.13-3.14					
**Contraceptive use at first sexual intercourse**			0.020	2.92	1.04-8.20	0.042	
No	1	1		1	1		
Yes	0.02	0.00-0.13	<0.001	0.01	0.01-0.30	0.007	
**Do you access contraceptives in your area**							
No	1	1		1	1		
Yes	2.37	1.07-5.28					
**Source of contraceptive knowledge†**			0.035	8.45	1.05-67.65		0.044
Mass media	1	1		1	1		
Family and friends	0.96	0.53-1.74	0.900	1.34	0.43-4.13	0.611	
Health professionals	0.23	0.11-0.48	<0.001	0.18	0.04-0.78	0.021	
Course education	0.30	0.08-1.11	0.070	2.32	0.30-18.00	0.421	

**†re-categorized** OR refers to odds ratio; AOR refers to adjusted odds ratio; CI refers to confidence interval and p refers to the p-value at p ≤ 0.05

All the variables with a p-value less than 0.15 at the bivariate level were retained for multivariate analysis. In the multivariate analysis, women who had never married, (AOR = 0.24, 95 % CI: 0.08, 0.75), frequency of contraceptive use (for occasional users: AOR = 0.01, 95 % CI: 0.00, 0.08; for regular users AOR = 0.01, 95% CI: 0.00, 0.04), use of contraceptives at first sexual intercourse (AOR = 95% CI: 0.03, 95% CI: 0.00, 0.42) and having history of abortion (AOR = 0.19, 95% CI: 0.05, 0.76) were found to have protective effect in intention to use contraceptives. On the contrary, urban residents (AOR = 6.40, 95% CI: 1.22, 24.66) and women with a history of unintended pregnancy (AOR = 95% CI: 1.02, 9.80) were more likely to have the intention to use contraceptives than their counterparts (Table 2). Moreover, women who had sex once a month had a 3.59 times greater intention to use contraceptives than women who had sex once a week (AOR = 3.59, 95% CI: 1.23, 10.53). The effect of ‘*source of contraceptive knowledge´ on ‘intention to use contraceptives’* was not significantly different among women who use mass media as compared to family and friends (AOR = 1.02, 95% CI: 0.34, 3.09) and course education (AOR = 2.17, 95%: 0.27, 17.67). Mass media as a source of knowledge had a significantly higher effect on the intention to use contraceptives as compared to that of health professionals (AOR = 0.21, 95% CI: 0.05, 0.85). However, age (p= 0.09), and attitude towards contraceptive use (p= 0.36) were not significantly associated with intention to use contraceptives.

## Discussion

The provision of modern family planning methods is essential for women in the postpartum period, as fertility can return unexpectedly and swiftly after giving birth if not breastfeeding [22,23]. In some cases, women have become pregnant before having their first menstruation following a delivery. Often, the pregnancy is unwanted and may end up being aborted [24]. This study was done to identify predictors of extended postpartum modern contraceptive use among women in urban Ghana. Results showed that the intention to use modern contraceptives after delivery was 30.20%. The oral contraceptive method (18.8%) was the main method preferred to be used by postpartum women, followed by a condom (2.3%), and an IUD (1%), respectively. Injection and implant would be the least used contraceptive methods at 0.7 %. This finding was similar to studies done in Ethiopia, Kenya, and Malawi [25-29]. On the other hand, the findings of this study are inconsistent with results from a similar study in Nairobi, Kenya, and Tigray Region, Northern Ethiopia, where the contraceptive use prevalence rates were 86.3% and 84.3%, respectively [30,31]. This high discrepancy may be due to different settings, improvement in health service delivery, the difference in the study period, and also the different socio-economic status of the study participants. Also, our study revealed that the odds ratios of using contraceptives were significantly lower among postpartum women who had never married compared to those who were married. This finding is similar to previous cross-sectional studies in Uganda and Addis Ababa, Ethiopia [32,33]. This may be ascribed to the fact that married women are mostly involved in unprotected sex as compared to unmarried women. Additionally, postpartum contraceptive use was found to be positively associated with women's residency. For instance, compared to those who lived in rural areas, the odds of using contraceptives were significantly higher among urban women. This finding is consistent with other similar studies in Uganda and Bangladesh [34,35]. We suggest that urban women may have been exposed to information from radios, magazines, the internet, posters, and televisions, as well as the availability of health centers to provide family planning services and information, compared to those in rural areas [35]. Also, urban women may easily have access to services due to the availability of health centers, while women residing in rural areas cannot, as they need to travel long distances to access such services [34,35]. Moreover, health professional workers have a bigger role to play, like helping clients to choose methods that suit them best and giving information that clients need for high-quality and effective family planning and care [36]. This study revealed that pregnant women who sourced contraceptive knowledge from professional health workers were 0.21 times less likely to use family planning methods compared to those who heard from mass media. This might be due to the approach by the health workers in communicating contraceptive information to women. This requires further study [37]. This is partly consistent with a report from Uganda, which showed that mass media had a positive impact on intention to use contraceptives [38].

Several studies have shown that better access to family planning services helps women to avoid unwanted pregnancies and plan birth spacing as they want [39]. This enables women and children to plan their educational and professional developments, family income, and stability. This study revealed that women who reported having easy access to contraceptives in their area of residence were 8.45 times more likely to use contraceptive methods than their counterparts. This corroborates a report from a similar study in Uganda [39]. Furthermore, family planning is not limited to controlling the number of persons in a family but also preventing pregnancy-related health threats in women and decreasing the need for unsafe abortion and infant mortality [12]. The study showed that pregnant women who had an abortion were less likely to use contraceptive methods than their counterparts. This might be due to misconceptions about family planning and abortion. This is similar to the report in Kenya [40]. Experience with family planning in terms of side effects has a great impact on intentions to use contraceptives [41]. The current study revealed that pregnant women who always use contraceptives had a higher intention to use contraceptive methods than those who had never used them before [31]. Our results corroborate other studies done in Ohio, the United States, and Sunyani, Ghana [42,43]. Our study revealed that women who engage in sexual activity once a month were 3.59 [AOR = 3.59, 95% CI: (1.23-10.53)] more likely to utilize contraceptives than their counterparts. The results indicate that married or cohabiting women who rarely engage in sexual intercourse might not want more children [44]. The sexual drive or urge of a woman decreases as the number of her children increases [45,46]. The present study showed that pregnant women who used contraceptives at their first sexual intercourse were 0.03 [AOR = 0.03, 95% CI: (0.00-0.24)] less likely to use contraceptives than their counterparts. This might be due to side effects and misconceptions from friends or partners [47]. These results corroborate reports by Chofakian *et al*. [48] in Brazil.

**Limitations:** the limitation includes the cross-sectional nature of the study, which prevents making any causal inference about the association. Also, information on contraceptive use was self-reported; hence, the chances of reporting bias cannot be ignored while interpreting the findings. Besides, the health facility-based population is limited and may not reflect the postpartum contraceptive use in other parts of Kumasi or other parts of Ghana when generalizing the findings. Future studies may increase the sample size and include husband/partner´s perspectives on extended postpartum contraceptive usage to better understand and identify predictors of postpartum contraceptive use in Ghana. In order to collect more credible and comprehensive information, both quantitative and qualitative methods may be used in future studies. The findings of the present study have important implications for policymaking. Having a high fertility rate and among the fastest-growing populations, the Ghanaian Government has displayed strong obligations to control population growth and advance reproductive healthcare services. Ghana also ranks high among the countries with high maternal mortality rates, which makes it imperative to increase research and development investments in family planning and other core maternal healthcare services. This would be very necessary if Ghana has to achieve the Sustainable Development Goal target of 70 per 100,000 live births in 2030. Evidence shows that the socio-economic gap in the use of maternal healthcare services has been decreasing slowly; however, substantially low contraceptive usage continues to persist. Our findings further support the need for strengthening political efforts, reproductive health advocacy, and counseling programs to resolve the geopolitical issues for promoting the use of family planning services in the country.

## Conclusion

The study has shown that postpartum contraceptive utilization was low (30.20%) among postpartum women in the study area in Ghana. Residing in urban areas, a history of unintended pregnancy, and sexual activity once a month were positively associated with postpartum contraceptive utilization. Conversely, postpartum women who sourced contraceptive knowledge from professional health workers, the unmarried, those with abortion history, postpartum women who frequently and occasionally use family planning, and women who used contraceptives at first sexual intercourse were negatively associated with postpartum contraceptive utilization. Health care workers should provide family planning counseling during the antenatal and postpartum period to educate, motivate, and inform women to make their own decisions and choices about postpartum family planning. Furthermore, community health education for pregnant women is important to prepare them for informed postpartum family planning decision-making. Contraceptive use education through the media from family planning professionals should be intensified to demystify the myths and misconceptions about contraceptives.

### What is known about this topic

- Modern contraceptives and intention to use contraceptives are concomitantly low, particularly in low-middle-income countries;
- Long-acting reversible contraceptives (LARCs) are the most effective reversible methods of contraception with a failure rate of less than 1%; thus, could prevent unplanned pregnancies post-partum.

### What this study adds

- The prevalence of post-partum modern contraceptive use intention was 30.20% among pregnant women;
- The most frequent method of intended use was oral contraceptives (18.8%);
- Urban residence, history of unintended pregnancy and frequency of sexual activity- once a month, are positively associated with post-partum contraceptive use intentions among pregnant women.
